# Examination of the Ovarian Reserve after Generation of Unilateral Rudimentary Uterine Horns in Rats

**DOI:** 10.1155/2014/918496

**Published:** 2014-02-06

**Authors:** Hasan Toyganözü, Hakan Nazik, Raziye Narin, Deniz Satar, Mehmet Ali Narin, Sinem Büyüknacar, Murat Api, Hakan Aytan

**Affiliations:** ^1^Department of Obstetrics and Gynecology, Adana Numune Education and Research Hospital, 01330 Adana, Turkey; ^2^Department of Pharmacology, Çukurova University Medical School, Adana, Turkey; ^3^Department of Obstetrics and Gynecology, Zeynep Kamil Education and Research Hospital, Istanbul, Turkey; ^4^Department of Obstetrics and Gynecology, Mersin University Medical School, Mersin, Turkey

## Abstract

*Objective*. The purpose of this experimental rat model study is to evaluate the changes in the ovarian environment after excision of the rudimentary horn. *Methods*. Ten female Wistar albino rats were used in this study. One cm of right uterine horn length was excised in the first operation. Two months after the first operation, all animals were sacrificed to obtain ovaries for histological examination. Mann-Whitney *U* test and Student's *t*-test were used for statistical analysis purposes. Statistical significance was defined as *P* < 0.005. *Results*. The number of primordial follicles (*P* = 0.415), primary follicles (*P* = 0.959), preantral follicles (*P* = 0.645), antral follicles (*P* = 0.328), and Graafian follicles (*P* = 0.721) was decreased and the number of atretic follicles (*P* = 0.374) increased in the right ovarian side. Howeve,r this difference was not found to be statistically significant. *Conclusion*. The results of this experimental rat model study suggest that the excision of rudimentary horn could have negative effects on ipsilateral ovarian functions.

## 1. Introduction

Congenital Müllerian anomalies account for the majority of uterus anomalies. The prevalence of uterine anomaly in the general population is estimated to be 0.5% [1]. Unicornuate uterus occurs in the event of arrest or failure of development of one Müllerian duct. The incidence of unicornuate uterus in uterine anomalies has been reported at 14% [2]. The classification of the American Society for Reproductive Medicine (ASRM) divides this anomaly group into four subgroups: cavity rudimentary horn communicating with the unicornuate uterus, noncommunicating cavity rudimentary horn, no-cavity rudimentary horn, and unicornuate uterus without rudimentary horn. A unicornuate uterus may lead to many gynecological or obstetric complications. They include infertility, dysmenorrhea, endometriosis, and premature birth [3, 4]. If pregnancy occurs in the rudimentary horn, then there is high risk of uterine rupture. So, it is suggested that the rudimentary horn is excised as soon as it is diagnosed due to such risks [4]. No sufficient data is available covering the effects of the excision of a rudimentary uterine horn that is on the ovarian reserve located on the same side. It has been previously shown that tubal ligation and hysterectomy affected the blood flow to the ovary and decreased the ovarian reserve [5–13].

Besides this, the diminished reserve may be due to some other factors such that uterus may have a mechanical supporting role to the ovary and removal may negatively affect this function which may consequently cause decrease in ovarian reserve. Another theory may be that there may be some unknown factors for blood supply or survival being transferred by contact with the uterus and removal may prevent this transfer. From here, we can say that theoretically the excision of rudimentary horn will likely negatively affect the ovarian reserve on the same side. The purpose of this experimental rat model study is to evaluate the changes in the ovarian environment after excision of the rudimentary horn.

## 2. Material and Method

This experimental study was conducted after obtaining the Ethical Committee Consent of the Experimental Medicine Research Institute, Faculty of Medicine, Çukurova University. The surgical arm of this study was conducted at the Animal Laboratory of the Department of Pharmacology, Çukurova University. And the histopathological morphometric analyses were made at the Pathology Laboratory of Adana Numune Education and Research Hospital. This study employed 10 female Wistar Albino rats weighing 180–220 grams. Adult female Wistar albino rats were housed in individual metabolic cages for two weeks to acclimatize them to the study surroundings.

All rats were anesthetized with ketamine hydrochloride (60 mg/kg IP) and xylazine hydrochloride (10 mg/kg IP). The abdominal skin was shaved and antisepsis was performed with a 10% povidone-iodine solution before the surgery. Using a sterile technique, a 3 cm vertical midline incision was performed and both uterine horns were exposed. About 1 cm of the right uterine horn was excised distally. The midline incision was closed in two layers by 2/0 polyglactin sutures: fascia and skin. The procedure was performed by two investigators. After the surgery, the rats were separately caged on the first day, and then all were kept in the same cage after making sure that they were healthy. A second laparotomy was performed 8 weeks after initial surgical procedure and the rats were sacrificed with high-dose (3 times the normal dose) ketamine anesthesia. Ovaries of the rats were excised. The ovaries were carefully separated from the surrounding fat layer and placed into a 10% formaldehyde solution for histopathological examination.

Tissues harvested from right and left ovaries for light microscopic examination were fixed in 10% buffered formalin solution. The materials were placed in cassettes, which were then put in a tissue processor. Paraffinized tissues removed from the tissue processor were then formed into blocks. A routine 4 *μ*m section was collected from the paraffin blocks, and one serial section in every 50 *μ*m was collected from each block, placed on a slide and stained with Hematoxylin and Eosin (H&E). H&E-stained preparations were examined using Olympus (Olympus BX53, Japan) light microscopy. Primordial (PrF), primary (PF), preantral, secondary, Graafian, atretic follicles and corpus luteum in each ovarian tissue were counted. The theca-interstitial cells in the ovarian stroma were evaluated. Their images were taken with an Olympus DP26 digital camera.

SPSS 11.5 (SPSS demo, Chicago, IL) software was used for statistical analysis. Kolmogorov-Smirnov test with Lillefor's correction was used to test whether the data used in the study were normally distributed. Normally distributed data were compared with a *t*-test used in two independent groups, and nonnormally distributed data was compared with the Mann-Whitney *U* test. Statistical significance was defined as *P* < 0.05.

## 3. Results

One of the 10 rats included in the study was lost due to anesthesia-related complications following the first surgery. One other rat was lost one week later after the operation. An examination showed that the rat died due to an infection. The ovarian follicle counts of the remaining 8 rats in the study and a comparison of the ovarian follicles on the side of the resection (right ovary) and the other ovary (left ovary) are shown in Table 1.

Primordial follicles, corpus lutea, and atretic follicles were compared using *t*-test as they were normally distributed, and the primary follicles, preantral follicles, secondary follicles, and Graafian follicles were evaluated using the Mann-Whitney *U* test as they were nonnormally distributed.

The number of primordial follicles (*P* = 0.415), primary follicles (*P* = 0.959), preantral follicles (*P* = 0.645), antral follicles (*P* = 0.328), and Graafian follicles (*P* = 0.721) was decreased and the number of atretic follicles (*P* = 0.374) increased in the right ovarian. However, this difference was not found to be statistically significant.

**Figure 1:**
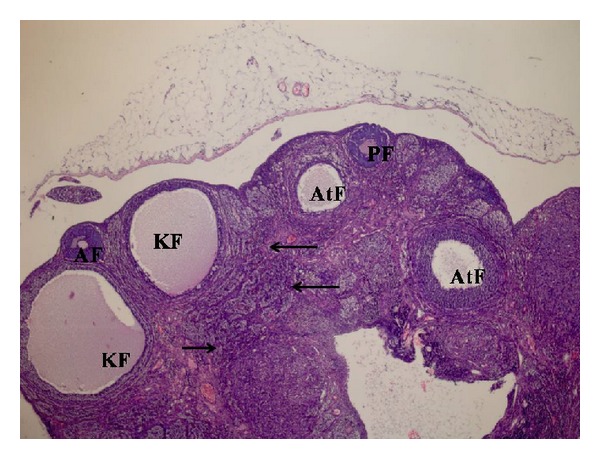
A slight decrease in primary follicles (PF) and antral follicles (AF) in right ovary sample, as well as an increase in theca interna cells in the more intensively observed cystic follicles (KF), atretic follicles (AtF), and ovarian stroma (arrows) (H&E ×40).

**Figure 2:**
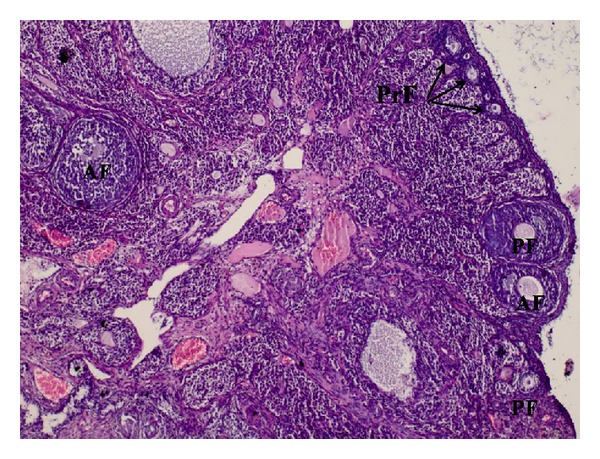
Ovarian tissue sample where all stages of follicular development containing primordial follicles (PrF-arrows), primary follicle (PF), and antral follicle (AF) are clearly seen in the left ovary sample (H&E ×40).

Images of the histopathological examination of right and left ovaries are given in the Figures 1 and 2. Increased atretic follicles, calcification, and fibrosis are seen in the right ovary. The right ovary also clearly shows reduced primary and antral follicles (Figure 1).

Section of the left ovary is seen in Figure 2. It is observed that all stages of follicular development containing primordial follicles, primary follicle, and antral follicle (AF) are clearly seen in the left ovary sample. The ovarian reserve is better compared with the right ovary (Figure 2).

## 4. Discussion

Women with unicornuate uterus suffer many obstetric and gynecological problems. Women with unicornuate uterus demonstrated higher rates of infertility, endometriosis, hematometra, and dysmenorrhea [3, 4]. As the probability of rupture and associated intra-abdominal bleeding risk is higher in the cavity rudimentary horn during pregnancy, this horn is recommended to be excised immediately after diagnosis [4].

There is insufficient data about the effects of the excision of the uterus rudimentary horn over the ovarian reserve on the same side. It has been previously demonstrated that tubal ligation and hysterectomy affected blood flow to the ovary and reduced the ovarian reserves [5–13]. From here, theoretically, it is likely that the excision of the rudimentary horn will also negatively affect the reserve of the ovary on the same side. The purpose of this study is to investigate whether the partial excision of the uterine horn affects the reserve of the ipsilateral ovary in a rat model. There is insufficient data covering the effects of the excision of the uterus rudimentary horn over the ovarian reserve on the same side. Previous studies investigated the effects of hysterectomy and tubal ligation over the ovarian reserve.

Among studies investigating the ovarian reserve after tubal ligation, elevated gonadotropin levels have been reported in some studies [5–7], whereas no changes have been reported by others [14–17]. Kelekci et al., in their prospective controlled study in 2006, have investigated ovarian reserve after tubal ligation. In this study, the main outcome measurements were blood levels of follicle-stimulating hormone (FSH), luteinizing hormone (LH), and estradiol (E_2_), ovarian volume, number of antral follicles and Doppler study of ovarian stromal artery pulsatile index (PI), and maximum velocity (V_max⁡_) on the third of the cycle immediately before and one and twelve months after the surgical intervention. A statistically meaningful increase was found in FSH and PI values after tubal ligation in the first month, but no statistically meaningful difference was found after twelve months. As a result, it was found that tubal ligation caused no differences in the ovarian reserve [18].

There is lack of consensus among studies investigating ovarian functions after hysterectomy. Some studies found that ovarian functions were affected [8–11], while others found the opposite; that is, they were not affected [19, 20]. Souza et al. investigated ovarian histology and function before and after total abdominal hysterectomy in 25 patients. Immediately before hysterectomy, bilateral ovarian biopsies were performed and, 12 months later, all patients underwent a second ovarian biopsy through laparoscopy. Histologic study of the ovaries one year after total abdominal hysterectomy showed stromal cell hyperplasia, thickening of the tunica albuginea, and a significant decrease of follicular reserve, follicular cysts, and corpora albicantia. There was no significant difference in the number of atretic follicles and corpora lutea. The serum levels of all hormones studied were unchanged 12 months after the surgical procedures [10].

Özdamar et al. investigated the ovarian morphology and FSH values on Wistar albino rats after hysterectomy. In their histological examination 6 months after hysterectomy, they reported that the ovarian cortex was fully covered with corpora lutea and the number of cystic follicles and atretic follicles increased, and they found only several secondary follicles and tertiary follicles, but no primary follicles. There is also a meaningful increase in FSH blood level [21]. In a similar study, Tapisiz et al. investigated the ovarian morphology and FSH, Inhibin A, and Inhibin B levels after hysterectomy on Wistar albino rats. Histologic examination of the ovaries was performed 100 days after hysterectomy and the findings were found to be similar to those of Özdamar et al. However, no significant change was found in hormonal levels [22].

Anti-Müllerian hormone is an important marker to show the ovarian reserve [23, 24]. Atabekoğlu et al. investigated the AMH levels after hysterectomy in order to evaluate ovarian reserves in 22 patients who underwent total abdominal hysterectomy. A reduction was found in AMH levels after hysterectomy, but it was statistically insignificant [25].

In this study, we created rudimentary horns in Wistar albino rats and then investigated the effects of the excision of rudimentary horn on the ovarian functions on the same side. Reviewing the literature revealed no similar studies. We found that the excision of rudimentary horn affected the particular ovary but this effect was statistically negligible. The possible mechanism might be the damaging of vascular structures passing to the ovary from the uterus due to the procedure and the reduction of blood flow to the ovary secondary to it.

Finding of no statistically meaningful difference between the two ovaries with regarding their reserves in our study might be due to our smaller number of cases. Thus, the study was launched with a total of 10 animals, and the results were based on 8 of them after loss of two animals. The minimum number of animals is recommended in studies on experimental animals. From here, exactly determination of whether or not the difference between the two groups is statistically meaningful can only be achieved through studies containing higher number of cases. So, studies with wider series are needed to cover the subject discussed here.

The most important limitation of this study is that, as in all studies on animals, the findings may not be accurately reflecting the effects on humans. In addition to the species difference between humans and animals, it should be noted that results can differ among members of the same species as well. This can be overcome by using inbred rats, but no inbred rats were used in this study due to inability to supply them.

As a result, we suggest the excision of the particular rudimentary horn in the event of potential complications in certain forms of the rudimentary horn anomaly. However, it is unclear how the ovarian reserve on the same side will be affected from this procedure at the end of this excision. This study shows that the excision procedure will negatively affect the ovarian reserve on the same side on a rat model. To the best of our knowledge, this is the first study that addresses this particular subject matter.

## Figures and Tables

**Table 1:** Comparison of the follicles in the right and left ovaries.

Ovary	Right side with resection		Left ovary		*P*
	Mean ± SD	Median (min–max)	Mean ± SD	Median (min–max)	
Primordial follicle	3.75 ± 3.41	2.5 (0–11)	5.37 ± 4.27	4.5 (1–15)	0.415
Primary follicle	0.87 ± 1.12	0.5 (0–3)	0.75 ± 0.7	1 (0–2)	0.959
Preantral follicle	2.5 ± 1.69	2 (0–5)	2.8 ± 1.72	2.5 (0–5)	0.645
Secondary follicle	0.75 ± 1.16	0 (0–3)	1.25 ± 0.88	1.5 (0–2)	0.328
Graafian follicle	0.12 ± 0.35	0 (0-1)	0.25 ± 0.46	0 (0-1)	0.721
Corpus luteum	4.3 ± 3.5	4 (0–10)	6.8 ± 2.5	6.5 (4–11)	0.129
Atretic follicle	3.0 ± 2.2	2.5 (0–6)	2.12 ± 1.55	1.5 (1–5)	0.374
